# Surgical management of upper limb lipoma arborescens: a systematic review

**DOI:** 10.1186/s13018-022-02997-7

**Published:** 2022-03-04

**Authors:** Georgios Kalifis, Nicola Maffulli, Filippo Migliorini, Theodorakys Marín Fermín, Jean Michel Hovsepian, Nikolaos Stefanou, Michael Hantes

**Affiliations:** 1 Department of Trauma and Orthopaedics, Hull Royal Infirmary, Anlaby Road, Hull, UK; 2 Department of Trauma and Orthopaedic Surgery, University Hospital of Larissa, Larissa, Greece; 3 Department of Medicine, Surgery and Dentistry, University of Salerno, Baronissi, Italy; 4 School of Pharmacy and Bioengineering, Keele University School of Medicine, Stoke on Trent, England; 5 Barts and the London School of Medicine and Dentistry, Centre for Sports and Exercise Medicine, Queen Mary University of London, Mile End Hospital, London, England; 6 Department of Orthopaedic, Trauma, and Reconstructive Surgery, RWTH Aachen University Hospital, Pauwelsstr. 30, 52074 Aachen, Germany; 7 Aspetar Orthopedic and Sports Medicine Hospital, Doha, Qatar; 8 Department of Sports Orthopaedics, Hessing Klinik, Augsburg, Germany

## Abstract

**Background:**

Lipoma arborescens (LA) is a rare benign synovial tumour characterized by the proliferation of mature adipocytes within the synovial cells. Given its rarity, current evidence is mainly based on case reports and case series, and no guidelines are available. The present study investigated the current surgical management and related outcomes of LA in the upper limb.

**Methods:**

This systematic review was conducted following the PRISMA guidelines. PubMed, Scopus, and Virtual Health Library were accessed in September 2021. Clinical studies evaluating patients with LA undergoing surgical treatment were considered eligible for this systematic review. Only studies which reported data on LA located in the upper limb with histopathological confirmation were considered. Articles that reported data from nonsurgical management were not considered.

**Results:**

A total of 21 studies reporting 22 lesions in 21 patients were assessed. The mean age of the patients was 48.48 years (range 22–77). Most studies evaluated the restoration of range of motion and symptom resolution for the functional outcome assessment. Open or arthroscopic excision and synovectomy were the most common surgical procedures for LA. The concomitant lesions were treated in a single-stage procedure. All patients had satisfactory outcomes after open or arthroscopic excision and synovectomy without recurrence at a mean follow-up of 21.14 months (range 2–60). One patient developed postoperative cellulitis (4.55%).

**Conclusion:**

Open and arthroscopic excision combined with synovectomy should be considered the standard treatment option of upper limb LA. Concomitant pathologies can be addressed in a one-stage procedure. Although LA was recognized as a clinical entity decades ago, there is a lack of evidence based guidelines and long term outcome data are unavailable.

## Introduction

Lipoma arborescens (LA) is a rare benign synovial tumour characterized by the proliferation of mature adipocytes within the synovial cells [1–5]. Clinical manifestations of LA are nonspecific and frequently resemble osteoarthritis, inflammatory arthritis, or infection [4, 6]. Monoarticular swelling or pain of insidious onset, intermittent joint effusion episodes or a slowly growing subcutaneous mass are common in patients with LA [1, 7]. Magnetic resonance imaging (MRI), using fat suppression or short tau inversion recovery (STIR) sequences point to the diagnosis in most patients with LA [8]. Although its etiology remains unknown [1], it has been hypothesized that LA may result from reactive differentiation of synovial cells towards adipocytes [9]. Two aetiological types have been described. The primary type is considered idiopathic and is mainly observed in younger population [7, 10, 11]. The secondary type is more common in the elderlies, and is associated with pathological conditions or lesions causing chronic irritation [7, 12]. The knee is the most frequent location of LA [1–3]; however, lesions of the wrist, elbow, shoulder, ankle, and hip joints have been reported [2, 10, 13–16]. For LA in the knee, arthroscopic synovectomy demonstrated excellent short-term results and a low rate of recurrence [15]. To the best of our knowledges, no review is available concerning the management of LA located in the upper limb. Given its rarity, current evidence is mainly based on case reports and case series, and no guidelines are available. The present study investigated the current surgical management and related outcomes of LA in the upper limb.

## Methods

### Search strategy

This systematic review was conducted following the Preferred Reporting Items for Systematic Reviews and Meta-Analyses (PRISMA) guidelines. Two investigators (G.K., TMF) independently performed the database search. PubMed, Scopus, and Virtual Health Library were accessed in September 2021. The terms "lipoma arborescens" AND/OR "synovial lipomatosis" AND/OR "villous lipomatous" were used alone and in combination (Additional file 1).

### Eligibility criteria

Clinical studies evaluating patients with LA undergoing surgical treatment were considered eligible for this systematic review. Given the authors language capabilities, articles published in English or Spanish were eligible. Only studies which reported data on LA located in the upper limb with histopathological confirmation were considered. Screening of the bibliographies of the potentially eligible articles was also performed. Articles that no clearly stated the length of the follow-up were excluded, as were those that did not report quantitative data. Articles that reported data from nonsurgical management were not considered.

### Data extraction and outcomes of interest

Two investigators (G.K., TMF) independently reviewed the included studies, and data were extracted to a predefined Excel spreadsheet with the following variables: author, year, type of study, number of women and mean age, history of inflammatory disease and trauma, number and location of the lesions, imaging studies, surgical procedures, length of the follow-up, recurrence, postoperative outcomes.

### Methodological quality assessment

The quantitative content assessment was performed using Murad's tool for evaluating the methodological quality of case reports and case series, which is a modified version of the Newcastle–Ottawa Scale [17]. This scale has been used recently in systematic reviews of case reports and case series [18–21]. The tool has five questions with dichotomic answers. A good assessment has to have five points, moderate four, and low less than three points.

### Statistical analysis

Data was presented in tables using absolute values from individual studies. Pooled data were presented as means with standard deviations and percentages. Statistical analysis was performed using SPSS V.19 and Microsoft Excel 2016 (Microsoft®, USA).

## Results

### Search results

**Fig. 1:**
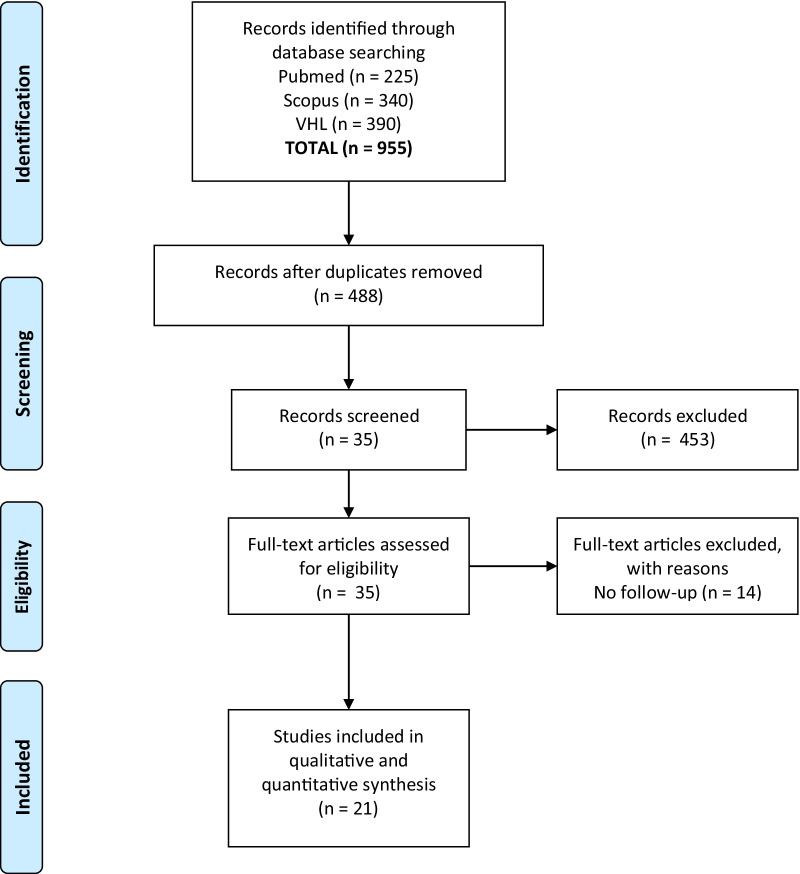
Flow chart of the literature search

The literature search identified 488 potentially relevant records after the exclusion of duplicates (*N* = 188). Titles and abstracts were screened and 35 articles were retrieved for full-text evaluation. No additional study was identified after citation screening. After full text assessment 14 studies were excluded due to insufficient data regarding follow-up. Twenty-one studies met the predetermined eligibility criteria (Fig. 1).

### Methodological quality assessment

The quality assessment was moderate for eight studies and low for 13. No single study was scored as good according to the modification of Murad et al. [17] (Table 1).

**Table 1:** Outcomes of Murad’s tool for methodological qualities assessment of case reports and case series [(1) Did the patient(s) represent the whole case(s) of the medical center? (2) Was the diagnosis correctly made? (3) Were other important diagnosis excluded? (4) Were all important data cited in the report? (5) Was the outcome correctly ascertained?]

Studies	1	2	3	4	5	Assessment
Nisolle et al. [22]	Yes	Yes	Yes	Yes	No	Moderate
Levadoux et al. [23]	Yes	Yes	Yes	Yes	No	Moderate
Kaneko et al. [24]	Yes	Yes	No	Yes	No	Low
Doyle et al. [25]	Yes	Yes	Yes	Yes	No	Moderate
Dinauer et al. [26]	Yes	Yes	No	Yes	No	Low
Yildiz et al. [27]	Yes	Yes	No	No	No	Low
In et al. [28]	Yes	Yes	No	Yes	No	Low
Mayayo Sinués et al. [29]	Yes	Yes	No	No	No	Low
Chae et al. [30]	Yes	Yes	Yes	Yes	No	Moderate
Hill et al. [37]	Yes	Yes	No	Yes	No	Low
Benegas et al. [1]	Yes	Yes	Yes	Yes	No	Moderate
Silva et al. [31]	Yes	Yes	Yes	Yes	No	Moderate
White et al. [32]	Yes	Yes	No	Yes	No	Moderate
Kim et al. [33]	Yes	Yes	No	Yes	No	Low
Stepan et al. [14]	Yes	Yes	No	Yes	No	Low
Mohammad et al. [34]	Yes	Yes	Yes	Yes	No	Moderate
Beyth and Safran [2]	Yes	Yes	No	Yes	No	Low
Lim et al. [35]	Yes	Yes	No	Yes	No	Low
Paccaud and Cunningham [13]	Yes	Yes	No	Yes	Yes	Moderate
Kawashima et al. [3]	Yes	Yes	No	Yes	No	Low
Elamin et al. [36]	Yes	Yes	No	Yes	No	Low

### Synthesis of results

A total of 21 studies reporting 22 lesions in 21 patients were assessed. The patient demographics is summarized in Table 2. Twelve patients (57.14%) were men and 11 (42.86%) women. The mean age of the patients was 48.48 ± 15.98 years (range 22–77). Fourteen lesions were right-sided, three patients had a history of inflammatory disease, and three had a history of previous trauma.

**Table 2:** Patients demographics

Study	Sex	Age	Side	History of inflammatory disease	History of trauma
Elamin et al. [36]	F	55	L	No	No
Kawashima et al. [3]	M	67	L	No	No
Paccaud and Cunningham [13]	M	54	R	Rheumatoid arthritis	Not disclosed
Lim et al. [35]	F	38	R	No	Yes
Beyth and Safran [2]	M	44	R	Not disclosed	No
Mohammad et al. [34]	F	68	R	No disclosed	Not disclosed
Kim et al. [33]	F	43	R	Not disclosed	No
Stepan et al. [14]	F	24	R	No	Not disclosed
White et al. [32]	M	64	L	No	Not disclosed
Benegas et al. [1]	M	65	R	No	No
Hill et al. [37]	M	41	R	Not disclosed	Yes
Silva et al. [31]	M	45	L	No	Not disclosed
Chae et al. [30]	M	37	R	No	No
Mayayo Sinues et al. [29]	F	44	L	No	No
In et al. [28]	M	22	L	No	No
Yildiz et al. [27]	M	23	R	Not disclosed	No
Dinauer et al. [26]	M	37	R	No	Not disclosed
Doyle et al. [25]	F	50	L	Psoriatic arthritis	Yes
Kaneko et al. [24]	F	77	L	No	No
Levadoux et al. [23]	F	76	R	Psoriatic arthritis	No
Nisolle et al. [22]	M	44	R	No	No

Imaging findings and surgical treatment outcomes are summarized in Table 3. All patients had single lesion; one has a bilateral presentation [26]. Eleven lesions (50%) were located in the shoulder [1–3, 22, 24, 28, 30, 32, 33, 35, 36], seven (31.82%) in the elbow [13, 23, 25, 26, 29, 34], and four (18.18%) in the wrist [14, 27, 31, 37]. All patients but one had preoperative MRI scans during the diagnostic assessment [27]. Concomitant rotator cuff tears were reported in five patients [1, 3, 24, 35, 38]. Similarly, a labral tear [33], a long head biceps tendon fraying [32], and a distal biceps pathology [34] were concomitant lesions to the LA. Most studies evaluated the restoration of range of motion and symptom resolution for the functional outcome assessment. In one study [13], the Mayo Elbow Performance Score and Single Assessment Numeric Evaluation were employed. Open or arthroscopic excision and synovectomy were the most common surgical procedures for LA. The concomitant lesions were treated in a single-stage procedure. All patients had satisfactory outcomes after open or arthroscopic excision and synovectomy without recurrence at a mean follow-up of 21.14 ± 18.38 months (range 2–60). One patient developed postoperative cellulitis (4.55%) [37].

**Table 3:** Main findings

Study	Number of lesions	Location	Imaging studies	Procedure	Follow-up	Recurrence	Postoperative outcomes
Elamin et al. [36]	1	Shoulder (subacromial)	Xray: no	Arthroscopic excision	60	No	Full active ROM and normal RC strength
			MRI: supraspinatus tendinopathy with a partial tear. Soft tissue mass in the subacromial space measuring 2.5 × 1.0 × 0.5 cm				
Kawashima et al. [3]	1	Shoulder (subdeltoid)	Xray: normal	Arthroscopic synovectomy and RC repair	9	No	Occasional aching, good function
			MRI: subdeltoid fluid villous projections, full-thickness supraspinatus tear				
Paccaud and Cunningham [13]	1	Elbow (intraarticular)	Xray: no	Arthroscopic synovectomy and posterior humeroulnar decompression	14	No	Full ROM. Asymptomatic
			MRI: large intra-articular multilobulated pseudo-tumoral mass causing posterior humeroulnar impingement with mixed components including lipomatous and synovial fringes				
Lim et al. [35]	1	Shoulder (subacromial, subdeltoid)	Xray: bony spurs in the acromion and greater tuberosity	Arthroscopic bursectomy, lipoma excision, acromioplasty, and RC repair	5	No	Asymptomatic
			MRI: Partial-thickness bursal tear of the supraspinatus tendon, subacromial-subdeltoid bursa fluid-distended-fat like nodular projections, greater tuberosity, and lateral acromion osteophytes				
Beyth and Safran [2]	1	Shoulder (intraarticular)	Xray: Hill Sachs	Arthroscopic synovectomy and lipoma excision	12	No	Full ROM. Asymptomatic
			MRI: joint effusion and synovial hyperplasia				
Mohammad et al. [34]	1	Elbow (antecubital fossa)	Xray: reactive changes in the radial tuberosity	Open bicipitoradial bursectomy, lipoma excision, and biceps debridement	6	No	Occasional aching, no calcifications
			MRI: cystic swelling in the right bicipitoradial bursa with peripheral frond-like and ovoid fatty components. Thickening of the distal biceps tendon insertion and hypertrophy of the bicipital radial tuberosity with some associated edema and chronic bicipitoradial bursitis				
Kim et al. [33]	1	Shoulder (subdeltoid, subacromial)	Xray: multiple calcifications, enthesophyte at greater tuberosity	Open lipoma excision, lipoma arborescens excision, and arthroscopic posterior labrum repair	36	No	High satisfaction and no limitations
			MRI: paralabral cyst which extends into suprascapular and spinoglenoid notch after a posterior labral tear, SLAP, lipoma in front of the anterolateral cortex of the humeral head, encapsulated mass between infraspinatus and deltoid muscle, villous projections (lipoma arborescens) within the mass with osteochondral metaplasia				
Stepan et al. [14]	1	Wrist (dorsal-extensor retinaculum)	Xray: mass dorsal to the carpus, soft tissue, and fat attenuation	Open tenosynovectomy of the fourth dorsal compartment and fatty mass excision	3	No	Pain-free full shoulder function
			MRI: proliferative tenosynovitis distending the fourth dorsal compartment, containing extensive areas of thick, enhancing tenosynovium as well as macroscopic lobules of subsynovial fat encircling extensor digitorum communis and extensor indicis tendons				
White et al. [32]	1	Shoulder (bicipital groove)	Xray: normal	Open synovectomy, lipoma excision, tenodesis, diagnostic arthroscopy	6	No	Pain-free with full shoulder function and rotation
			MRI: frond-like tissue extending from the synovium, which followed the signal intensity of subcutaneous fat on all sequences. The synovium of the glenohumeral joint had no evidence of involvement by this process				
Benegas et al. [1]	1	Shoulder (intrarticular, subacromial)	Xray: increased soft tissue. Simple radiography did not show any abnormalities, except for increased soft-tissue volume	Arthroscopic and open synovectomy, lipoma excision, and RC repair	4	No	Asymptomatic
			MRI: full-thickness tear of the anterior portion of the supraspinatus tendon and significant glenohumeral and subacromial synovitis, with signs of fatty metaplasia				
Hill et al. [37]	1	Wrist (dorsal-extensor retinaculum)	Xray: dorsal soft-tissue mass-mild degenerative disease of the radioscaphoid joint	Open lipoma excisional biopsy	2	No	Significant improvement. Complication: minor postoperative cellulitis
			MRI: high signal intensity soft tissue lesion consistent with fat and multiple frond-like projections of similar intensity investing the extensor tendons				
Silva et al. [31]	1	Wrist (dorsal-extensor retinaculum, also in the knee and ankle)	Xray: soft tissue mass	Open excision	48	No	Asymptomatic
Chae et al. [30]	1	Shoulder (intraarticular)	Xray: humeral head erosion	Open synovectomy and lipoma excision	12	No	Favorable outcome
			MRI: well-capsulated, mass-like projections were encircling the right glenohumeral joint and containing a villonodular fat component				
Mayayo Sinues et al. [29]	1	Elbow (antecubital fossa)	Xray: soft tissue mass	Open partial synovectomy	48	No	Full ROM
			MRI: circumscribed mass along the bicipitoradial bursa enveloped the biceps tendon, with a heterogeneous signal bursal effusion and fat tissue deposits similar to small polypoid lesions from the wall to the interior of the mass				
In et al. [28]	1	Shoulder (intraarticular)	Xray: osteopenia and arthritic changes	Arthroscopic synovectomy	12	No	Uneventful
			MRI: intra-articular frond-like or villous nodules of high signal intensity represent fat. Bone erosion was present at the superior aspect of the humerus				
Yildiz et al. [27]	1	Wrist (dorsal-extensor retinaculum)	Xray: soft tissue mass	Open excision	24	No	Asymptomatic
Dinauer et al. [26]	2 (asynchronous bilateral lesion)	Elbow (bicipitoradial bursa)	Xray: a) normal; b) soft tissue swelling	Open excisional biopsy	a) 46; b) 6	No	Good function
			MRI: diffuse frond-like, fat-containing lesion involving the bicipitoradial bursa, lipoma arborescens arising from the bicipitoradial bursa was offered				
Doyle et al. [25]	1	Elbow (antecubital fossa)	Xray: no	Open excisional biopsy	12	No	Diminished pain
			MRI: lobulated mostly fatty mass anterior to the elbow joint and wrapping around the distal biceps tendon				
Kaneko et al. [24]	1	Shoulder (subdeltoid)	Xray: increased soft tissue	Open excisional biopsy and supraspinatus tear open repair	40	No	Full ROM. Residual pain
			MRI: villous mass with surrounding synovial fluid in sub-deltoid bursa FT tear ST. Enormous bursa				
Levadoux et al. [23]	1	Elbow (anterolateral mass)	Xray: normal	Open excisional biopsy	48	No	Full ROM. Asymptomatic
			MRI: joint effusion and synovial-based soft tissue mass. Numerous frond-like projections				
Nisolle et al. [22]	1	Shoulder (subdeltoid, subacromial)	Xray: soft tissue swelling	Open bursectomy and RC repair	12	No	Full ROM. Diminished pain
			MRI: full-thickness tear of the supraspinatus tendon and a large effusion within the bursa containing numerous frond-like projections				

## Discussion

According to the main finding of the present systematic review, patients undergoing surgical excision and synovectomy for LA of the upper limb evidenced satisfactory outcomes regardless of the surgical technique used, with low complication rate and no recurrences at approximately 2 years follow-up.

The aetiology of LA is still controversial. The present systematic review findings did not show a relevant correlation with either inflammatory disease or trauma history. Oni et al. [39, 40] suggested that LA may result from chronic synovitis, and questioned the lesion's pathognomonic findings found on MRI. On the other hand, Ragab et al. [41] suggested that LA may cause joint inflammatory synovitis, mimicking undifferentiated inflammatory arthritis. The authors highlighted the importance of diagnostic tools such as MRI that led to better decision-making and avoidance of unnecessary disease-modifying anti-rheumatic drug prescription [41]. Both theories regarding the aetiopathology of LA concluded that the lesion is closely related to or affected by inflammatory condition. Combining this chronic inflammation with mechanical irritation from the LA mass may predispose patients to other local concomitant lesions.

LA is characterized by typical pathognomonic MRI features. Frond-like architecture synovial mass with fat signal intensity in all sequences and suppression in short tau inversion recovery sequencing or spin-echo, associated with effusion, chemical-shift artifacts at the fat fluid interface without haemosiderin magnetic susceptibility effects, or intravenous contrast enhancement point toward LA. Specific features of the LA may provide useful information and may lead to better management [42, 43]. The included studies in the present systematic review suggested that LA may be present in combination with other concomitant pathological conditions, highlighting the importance of MRI for diagnosis and preoperative planning.

In common with other rare clinical entities, the management of LA lacks evidence-based guidelines. Being a benign lesion, theoretically, if asymptomatic, surgical intervention may not be mandatory [5]. However, to the best of our knowledge, there is no long-term follow-up study observing and examining the natural history of LA. Excision and synovectomy of the affected joint have been proposed as a treatment option. Both open and arthroscopic techniques have been reported, leading to good short-term functional results without recurrences [5, 15]. According to this systematic review, one-stage open or arthroscopic procedures address both LA and potential concurrent pathologies, such as rotator cuff or labral tears, and should be considered as standard treatment option.

This study has several limitations. The limited number of studies included for analysis and related sample size did not allow to infer solid conclusion. The length of the follow-up was limited in all the included studies. Moreover, there was a lack of validated tools in the outcome assessment. Finally, all of the studies included reported no recurrences, mainly based only on symptom regression. The limited length of the follow-up and the absence of imaging at the time of the final evaluation may have under-reported possible recurrences. Given the limited data available for inclusion, comparisons between open and arthroscopic management were not possible to evaluate. However, it is unclear whether lesion size and location may play a role in determining specific approaches. A systematic review on the arthroscopic treatment of LA of the knee revealed a satisfactory short-term outcome [15]. The present study supports similar findings: patients may benefit from less invasive arthroscopic procedures when feasible, as arthroscopic treatment of shoulder [2, 3, 28, 35, 36] and elbow lesions [13] led to promising short-term outcomes. Although LA was recognized as a clinical entity decades ago, the evidence is scarce and long term outcome data are unavailable.

## Conclusion

Open and arthroscopic excision combined with synovectomy should be considered the standard treatment option of upper limb LA. Concomitant pathologies can be addressed in a one-stage procedure. Although LA was recognized as a clinical entity decades ago, there is a lack of evidence based guidelines and long term outcome data are unavailable.

## Data Availability

All data generated or analysed during this study are included in this published article.

## Abbreviations

LA: Lipoma arborescens
MRI: Magnetic resonance imaging
STIR: Short tau inversion recovery
